# Cytomembrane‐Mediated Transport of Metal Ions with Biological Specificity

**DOI:** 10.1002/advs.201900835

**Published:** 2019-07-01

**Authors:** Ming‐Kang Zhang, Jing‐Jie Ye, Chu‐Xin Li, Yu Xia, Zi‐Yang Wang, Jun Feng, Xian‐Zheng Zhang

**Affiliations:** ^1^ Key Laboratory of Biomedical Polymers of Ministry of Education & Department of Chemistry Wuhan University Wuhan 430072 P.R. China

**Keywords:** bioimaging, biotargeted transport, cell membrane, metal ions, tumor therapy

## Abstract

Metal ions are of significant importance in biomedical science. This study reports a new concept of cytomembrane‐mediated biospecific transport of metal ions without using any other materials. For the first time, cytomembranes are exploited for two‐step conjugation with metal ions to provide hybrid nanomaterials. The innate biofunction of cell membranes renders the hybrids with superior advantages over common vehicles for metal ions, including excellent biocompatibility, low immunogenic risk, and particularly specific biotargeting functionality. As a proof‐of‐concept demonstration, cancer cell membranes are used for in vivo delivery of various metal ions, including ruthenium, europium, iron, and manganese, providing a series of tumor‐targeted nanohybrids capable of photothermal therapy/imaging, magnetic resonance imaging, photoacoustic imaging, and fluorescence imaging with improved performances. In addition, the special structure of the cell membrane allows easy accommodation of small‐molecular agents within the nanohybrids for effective chemotherapy. This study provides a new class of metal‐ion‐included nanomaterials with versatile biofunctions and offers a novel solution to address the important challenge in the field of in vivo targeted delivery of metal ions.

A number of metal ions play crucial roles in biological processes, such as the regulation over enzyme activation, inflammatory response, physiological behavior of cells, and protein synthesis. Therefore, metal ions have found increasingly important applications in biomedical fields, including disease therapy,^1^, ^2^ drug delivery,^3^ imaging diagnosis,^4^ biocatalysis,^5^ biosensor,^6^ artificial muscle,^7^ and so on. For instance, platinum (Pt) compounds are among the most widely used clinical agents for treating advanced cancers.^8^ Clinically, metal ions take prevalent roles as diagnostic agents, represented by the metal radionuclides in CT imaging and the paramagnetic metal complexes as contrast agents in magnetic resonance imaging (MRI).^9^, ^10^

To address the limitation of small molecule metal compounds that suffer from short circulation duration in vivo and high acute toxicity,^11^ second generation of metal ion–polymer composites have been intensively explored.^12^ Nevertheless, exotic materials would inevitably induce unwanted immune response, and up to date, the fate of the majority of man‐made polymers remains ambiguous yet.^13^ This dilemma inspires researchers to embark on the transport of metal ions using biomacromolecules coming from life systems, such as the recently highlighted proteins and genes.^14^, ^15^ Unfortunately, these biomacromolecules fail to find their way to the targeted sites when in vivo administrated, leading to significant concerns, for example, systematic toxicity and drug tolerance of patients.^16^ To make the biomacromolecules more suitable for in vivo delivery of metal ions, surface modification with targeting moieties (e.g., ligand and monoclonal antibody) has to be needed. However, the clinical promise of this strategy is highly downgraded by the complicated chemistry involved in the preparation process,^14^, ^17^ owing to the low reaction controllability that frequently causes polymer crosslinking, protein denaturalization, and so on.^18^ Therefore, an appealing challenge naturally arises that how to accomplish biospecific transport of metal ions while excluding exotic materials for high biosafety.

For the first time, the present study put forward a novel solution to this challenge by using the natural bioassembly of cell membranes for delivering metal ions. As a ubiquitously biological phenomenon, many cell lines display inherent tropism to specific tissues.^19^ For instance, macrophage is among the first population of immune cells to arrive at the sites of wounding and inflammation.^20^ Bleeding lesion would evoke the recruitment of platelet nearby.^21^ In animal models with myocardial infarction, myocardial homing of transplanted stem and progenitor cells has been identified.^22^ Also, cancer cells tend to aggregate together beside the blood vessel endothelium for the progression into tumor spheroids, known as “homologous adhesion” of cancer cells.^23^, ^24^ Although the underlying mechanisms are far from clear, the surface molecules on cell membrane are believed to play a crucial role in these biological actions.^25^ For instance, the homologous interaction between cancer cells is generally thought to have association with the expression of special cellular adhesion molecules (epithelial cell adhesion molecule, N‐cadherin, galectin‐3, etc.) on cancer cell membranes (CCMs).^23^ Such a unique biological nature evokes our interest to use cytomembranes to realize specific delivery of metal ions for medical applications without using any other materials. If this is accessible, we would offer a new concept of cytomembrane‐mediated transport of metal ions with versatile biofunctions that are hardly achieved by man‐made materials. Because the negatively charged cell membranes consist of bioengineered phospholipid bilayer embedded with vast proteins, cell membranes could readily conjugate with metal ions, thus offering a biomimetic identity for the in vivo transport process. As a proof of concept, we herein demonstrated the special role of CCM in the in vivo tumor‐specific delivery of metal ions, owing to the biological specificity of CCMs to homologous tumors.^10^, ^24^, ^26^ Based on different metal ions, such as ruthenium, europium, iron, and manganese, the formed hybrid nanomaterials showed favorable biospecific imaging/therapy potentials (**Scheme** 1).

**Scheme 1:**
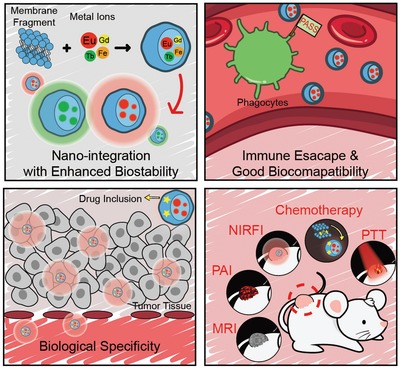
Illustration of hybrid metal ion/cell membrane materials—biomimic transport of metal ion with biological specificity.

Cell membrane cracks from mouse breast cancer (4T1) cells (termed CCMCs unless otherwise mentioned) were collected by the membrane protein extraction kit as we reported.^10^, ^24^, ^26^ The lyophilized CCMCs were dispersed in deionized water by an ultrasonic instrument. CCMCs were demonstrated to be able to conjugate readily with metal ions when ferric ion was used as the typical model. The two‐step preparation of the hybrid nanomaterials was briefly described as follows. Ferric chloride (10 mg mL^–1^) was added dropwise into CCMCs dispersion (1 mg mL^–1^). With the increasing dose of Fe^3+^, the zeta potential gradually rose and approximated to zero at the weight ratio of around 19:100 (Figure S1, Supporting Information). At this ratio, the formed particles were susceptible to sedimentation. When the ratio was further increased to 20:100, the zeta potential reached about +8.7 mV that was enough to stabilize the particles owing to the charge repulsion. Therefore, we chose this mass ratio for the following study. Centrifugation at 12 000 rpm was performed to isolate the positively charged particles (MFe), which then acted as a core for the second round of cytomembrane coating to enable the outer shell, which was entirely composed of cytomembranes. Specifically, MFe redispersed in deionized water was slowly added to CCMC dispersion under mild vortexing and then the mixture solution was physically extruded by an Avanti mini extruder. The hybrid particles, as termed MFe@M, were finally obtained by centrifuging at the optimized speed and washing repeatedly. As shown by transmission electron microscopy (TEM), MFe@M particles presented a compact nanostructure (**Figure** 1A). The hydrodynamic size of the MFe@M was about 120 nm, close to that of MFe (Figure S2A, Supporting Information). Upon the complexation, the zeta potential dropped from +8.7 mV (MFe) down to –26.2 mV (MFe@M) (Figure S2B, Supporting Information). There appeared no evident changes of the hydrodynamic size during a 7‐day period after MFe@M was transferred into PBS solution or the cell culture medium, indicating the excellent stability of MFe@M (Figure S3, Supporting Information). In contrast, MFe nanoparticles were prone to aggregation in two media, as reflected by the sharp size increase to the micron magnitude (Figure S4, Supporting Information). Quantitative analysis by inductively coupled plasma atomic emission spectroscopy showed that the iron content was 7.30 ± 0.41% in MFe and 6.39 ± 0.16% in MFe@M nanoparticles. The integration of ferric ion with CCMCs led to a significant enhancement of UV–Vis absorbance (Figure 1B) and an evident change of solution color (Figure 1C1). The same was true when other metal ions were applied (Figure S5, Supporting Information). After the high‐speed centrifugation to remove MFe@M nanoparticles (Figure 1C2), ferric ion was hardly detectable in the supernatant by Fehling reagent (Figure 1C3). These results manifested that there indeed happened strong interaction between metal ions and CCMCs.

**Figure 1:**
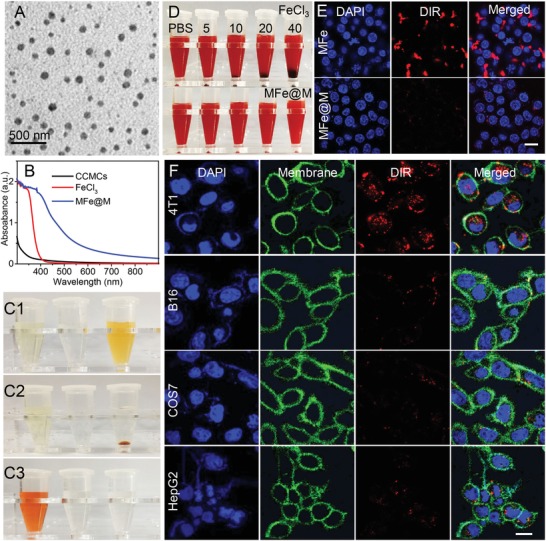
A) TEM images of MFe@M nanoparticles. B) UV–Vis spectra of CCMCs, FeCl_3_, and MFe@M. C) Photographs of FeCl_3_, CCMCs, and MFe@M before (C1) and after (C2) centrifugation and C3) photo images of supernatant of FeCl_3_, CCMCs, and MFe@M solutions after the centrifuging followed by the addition of Fehling reagent. D) Observation over the solution of RBC cells after the addition of FeCl_3_ and MFe@M. Concentrations of Fe^3+^ were 5, 10, 20, and 40 μg mL^−1^. E) Cellular internalization by macrophages after co‐incubation with MFe and MFe@M for 4 h. F) Cellular internalization assay of MFe@M‐4T1 in 4T1, B16, COS7, and HepG2 cells. The nucleus was stained blue with DAPI, the cell membrane was stained green with cell mask green, and MFe@M‐4T1 was stained red with DIR. Scale bar: 20 µm.

The delicately engineered MFe@M nanoparticles are expected to share the biological merits of parent CCMs, including innate biocompatibility, evasion from autologous immune, and specific affinity with homologous tumors, which completely match the rigid requirement for effective and safe delivery in vivo. As expected, MFe@M exhibited excellent biocompatibility with red blood cells and caused minimal coagulation (Figure 1D) and hemolysis (Figure S6, Supporting Information). In comparison, the introduction of ferric ions at the identical concentration led to insignificant hemolysis but rapidly severe coagulation. Nearly no cell‐toxic effects were found in NCTC 1649 (normal hepatocytes of mice) and 4T1 cells when they were exposed to MFe@M nanoparticles within the concentration range up to 400 µg mL^–1^ (Figure S7, Supporting Information). Furthermore, we roughly evaluated the in vivo toxicity toward liver and kidney after intravenous injection of MFe@M. In two weeks postinjection, main indicators reflecting the functions of liver and kidney, such as ALT (alanine aminotransferase), AST (aspartate aminotransferase), GGT (γ‐glutamyl transpeptidase), and urea, showed no evident deviations from the negative control administrated with PBS solution (Figure S8, Supporting Information). Also, the major indexes of blood routine examination remained steady after the injection of MFe@M (Figure S9, Supporting Information). All these results indicated the favorable biocompatibility of MFe@M to a great extent.

Foreign materials are easily recognized by macrophages and then eliminated, which is known as one of the important barriers responsible for their low bioavailability.^27^ Owing to the superficial location of CCM, MFe@M nanoparticles are thought to share the inherent ability of cancer cells to avoid phagocytosis. The MFe@M nanoparticles was labeled with 1,1‐dioctadecyl‐3,3,3,3‐tetramethylindotricarbocyaine (DIR), a near‐infrared (NIR) dye. As shown in Figure 1E, the fluorescently labeled MFe@M was actually barely detectable in macrophages when they were co‐incubated together for 4 h. In comparison, MFe nanoparticles, although including membrane fragments, were susceptible to cellular internalization by macrophages. The marked deviation was due to the enrichment of metal ions on MFe surface, reconfirming the advantage of our two‐step preparation pathway. Most tumor cells over‐express CD47 protein on their cytomembrane like a “don't eat me” signal to help themselves escape from macrophage capture (Figure S10, Supporting Information).^28^ However, apoptotic cancer cells are supposed to express “eat me” signals, such as phosphatidylserine, for the easy uptake by macrophages.[qv: 28c] CD47 protein and phosphatidylserine on cytomembranes were thus stained to compare the ratios in the mean fluorescence intensity among live 4T1 cells, MFe@M, and apoptotic 4T1 cells (Figure S10, Supporting Information). The ratios were calculated to be 12.75, 11.96, and 1.35, respectively. This result indicated that MFe@M prepared by our membrane isolation approach shared the macrophage‐evasion capability with live cancer cells to a large extent.

The specific targeting of MFe@M nanoparticles toward the homologous cancer cell and tumor tissue is pretty appealing because the targeted delivery of metal ions appears to be difficultly accessible owing to the tedious preparation of the vehicles. Cell uptake behaviors of the 4T1 cell membrane containing MFe@M‐4T1 were comparatively studied in 4T1, B16 (mouse melanoma cells), COS7 (African green monkey kidney fibroblasts), and HepG2 (human hepatoma carcinoma cells) cell lines by confocal laser scanning microscopy. After 4 h incubation, the red fluorescent signal with strong intensity appeared in 4T1 cells, but it was almost not detectable in the other tested cells (Figure 1F). The quantitative evidence of selective cell uptake was provided by flow cytometry (Figure S11, Supporting Information). The uptake efficiency in homologous 4T1 cells was much higher than that in the heterogeneous cells. Moreover, there were no significant differences in the uptake efficiency among those heterogeneous cells. It was evident that MFe@M had the specific affinity with homologous 4T1 cells. The in vitro homologous recognition in cellular levels encouraged us to investigate the in vivo biotargeting of MFe@M toward the homologous tumor developed from the same cancer cells. The DIR‐labeled samples including MFe@M‐4T1, MFe@M‐CT26 prepared from CT26 cell membranes, MFe, and CCMC‐4T1 were intravenously injected into the 4T1‐tumor‐bearing mice. The in vivo fluorescence images were obtained at the predetermined intervals by an animal living imaging system. As shown in **Figure** 2A–C, in the group of MFe@M‐4T1, fluorescence at the tumor site gradually increased within 72 h and remained at a high level for a long time. MFe@M‐4T1 exhibited superior advantage for tumor targeting over MFe and MFe@M‐CT26 nanoparticles. The fluorescence signal of MFe@M‐CT26 at 4T1 tumors was very weak, even lower than the positively charged MFe that contained 4T1 cell membrane yet without further membrane coating. This result reconfirmed the important role of homologous targeting from another aspect. It is reasonable that in comparison, CCMCs showed more evident tendency to accumulate at liver and spleen while being rarely distributed in tumors. This is mainly due to that the complexation with metal ions provided the compact nanoparticles, offering chances for the combination of passive and active targeting. In addition, the stretching morphology of free cytomembrane with a larger size should be taken into account in terms of the uptake deviation in different organs. Collectively, all the results proved the strong potency of MFe@M‐4T1 for the in vivo biotargeting at homologous 4T1 tumors. The membrane proteins contained in MFe@M were examined by SDS‐PEG gel electrophoresis, revealing that the membrane proteins were almost reserved in MFe@M (Figure S12, Supporting Information). All these results suggested that the combination of CCMCs with metal ions using our two‐step method affected insignificantly the biotargeting ability of cytomembranes. Of note, the complexation between metal ions and CCMCs into nanoparticles appeared to benefit the tumor‐targeting performance based on the comparison with the group of CCMCs alone. Without metal conjugation, CCMCs tended to accumulate at liver and spleen while being rarely distributed in tumors. The result might have association with the different metabolic pathway of free cytomembrane in addition to the membrane morphology, although the definite mechanism is unclear at present. Anyway, the results adumbrated that MFe@M may potentially act as a class of biologically derived probe for tumor detection.

**Figure 2:**
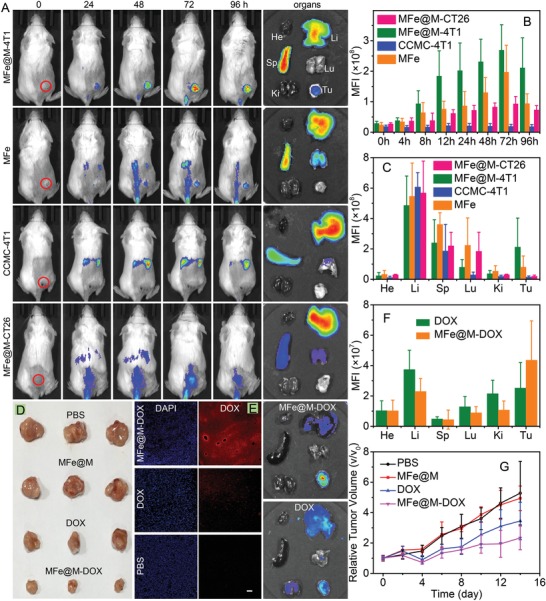
A) In vivo fluorescence images of 4T1‐tumor‐bearing mice and ex vivo fluorescence images of major organs after intravenously injected MFe@M‐4T1, MFe, CCMC‐4T1, and MFe@M‐CT26. B) Mean fluorescence intensity at tumor sites in the mice treated with MFe@M‐4T1, MFe, CCMC‐4T1, and MFe@M‐CT26. C) Mean fluorescence intensity of major organs in the mice treated with MFe@M‐4T1, MFe, CCMC‐4T1, and MFe@M‐CT26. (He, heart; Li, liver; Sp, spleen; Lu, lung; Ki, kidney; Tu, tumor). D) Photographs of tumors in different groups after treatment. E) Fluorescence images of tumor slices and photo images of major organs in the groups of MFe@M‐DOX and DOX. (Blue, DAPI; red, DOX) Scale bar: 50 µm. F) Mean fluorescence intensity of major organs in the groups of MFe@M‐DOX and DOX. G) Variation of relative tumor volume after different treatments.

Because of the phospholipid bilayer structure, cytomembrane has the potency to load hydrophobic drugs inside lipid layer.^29^ Doxorubicin (DOX), a broad‐spectrum antitumor drug, was loaded in MFe@M and the loading content can reach 14.2 wt%, much higher than that of many organic and inorganic carriers.^30^ MFe@M‐DOX exhibited no evident changes compared with MFe@M in both morphology and size (Figure S13, Supporting Information). The assay of DOX fluorescence in tumors after intravenous injection of DOX‐containing samples was shown in Figure 2E,F, indicating that MFe@M nanoparticles were more effective to deliver DOX to tumor sites compared to the injection with free DOX. Consequently, MFe@M elicited much stronger tumor‐inhibition effect (Figure 2D,G). The average weight of mice showed no significant differences among all the tested groups in 2 weeks (Figure S14, Supporting Information). H&E staining of normal organs indicated that the administration of MFe@M‐DOX caused minimal systematic toxicity (Figure S15, Supporting Information). These results suggested the potential of MFe@M as a new kind of nanoplatform for in vivo tumor‐specific drug delivery.

Next, other metal ions were chosen as models to demonstrate the advantages arising from the individual characters of metal ions. Recently, ruthenium‐based compounds have been developed with versatile biochemical properties.^31^ Ruthenium‐based compounds can be applied for photodynamic and photothermal therapies.^32^ Here, we prepared MRu@M nanoparticles in the same manner (Figure S16, Supporting Information). Upon complexation, the color of MRu@M solution became darker (Figure S17, Supporting Information). Correspondingly, MRu@M displayed stronger UV–Vis‐NIR absorption than its free form (Figure S6, Supporting Information). The complexation with CCMCs led to considerably enhanced photothermal conversion efficiency (**Figure** 3A), as also proved by the larger temperature increase detected by an infrared thermal imaging camera (Figure S18, Supporting Information). The photothermal conversion efficiency (η) of MRu@M was determined to be ∼37.2% (Figure 3B,C), which is higher than majority of nanomaterial‐based photothermal agents such as Au nanorods (21%),^33^ graphene oxide (25%),^34^ and Cu_2–*x*_Se nanoparticles (22%).^35^ In contrast, CCMCs alone failed to generate heat under irradiation. The robust ability of photothermal conversion stimulated us to explore the potential of MRu@M for in vivo photothermal imaging (PTI) and photothermal therapy (PTT). For direct comparison, 4T1 tumor bearing mice were randomly divide into six groups ready for the intratumoral injection with PBS, RuCl_3_, and MRu@M with or without NIR irradiation (0.8 W cm^−2^, 5 min) at 6 h postinjection. Thermal images revealed that tumor sites presented an apparently higher temperature than normal tissues in the groups of RuCl_3_+NIR and MRu@M+NIR (Figure 3E). Typically, the tumor temperature after RuCl_3_+NIR treatment was increased to around 45 °C within 1 min, while the temperature could be elevated up to 52 °C for MRu@M+NIR group at the same Ru^3+^ concentration of 100 µg mL^−1^ (Figure 3D). This temperature was documented to be sufficient for effective elimination of cancer cells.^36^ As shown in Figure 3G, MRu@M+NIR group exhibited apparently better antitumor efficacy than the other groups. Consequently, the tumors in MRu@M+NIR group were near to complete elimination (Figure 3H). Based on the results of the weight variation of treated mice (Figure 3F) and the H&E staining assay toward normal tissues (Figure S19, Supporting Information), it was suggested that the MRu@M‐induced PTT posed minimal side effects to normal organs. Photoacoustic imaging (PAI), a noninvasive imaging technology with high spatial resolution and deep imaging depth, has been widely applied.^37^, ^38^ MRu@M with strong absorption in the NIR region showed an excellent photoacoustic response. The intensity of the PA signal was positively proportional to MRu@M concentration (*R* ^2^ = 0.999) and MRu@M afforded a strong photoacoustic signal at a low concentration (Figure 3I and Figure S20, Supporting Information), and the extinction coefficient (ε) of MRu@M at 808 nm was calculated to be 3.1 × 10^8^, which was even superior to gold nanoparticles and small molecule dyes.^38^, ^39^ These results indicated that MRu@M may serve as a diagnostic agent for NIR‐mediated therapy/bioimaging, such as PTI, PTT, and PAI.

**Figure 3:**
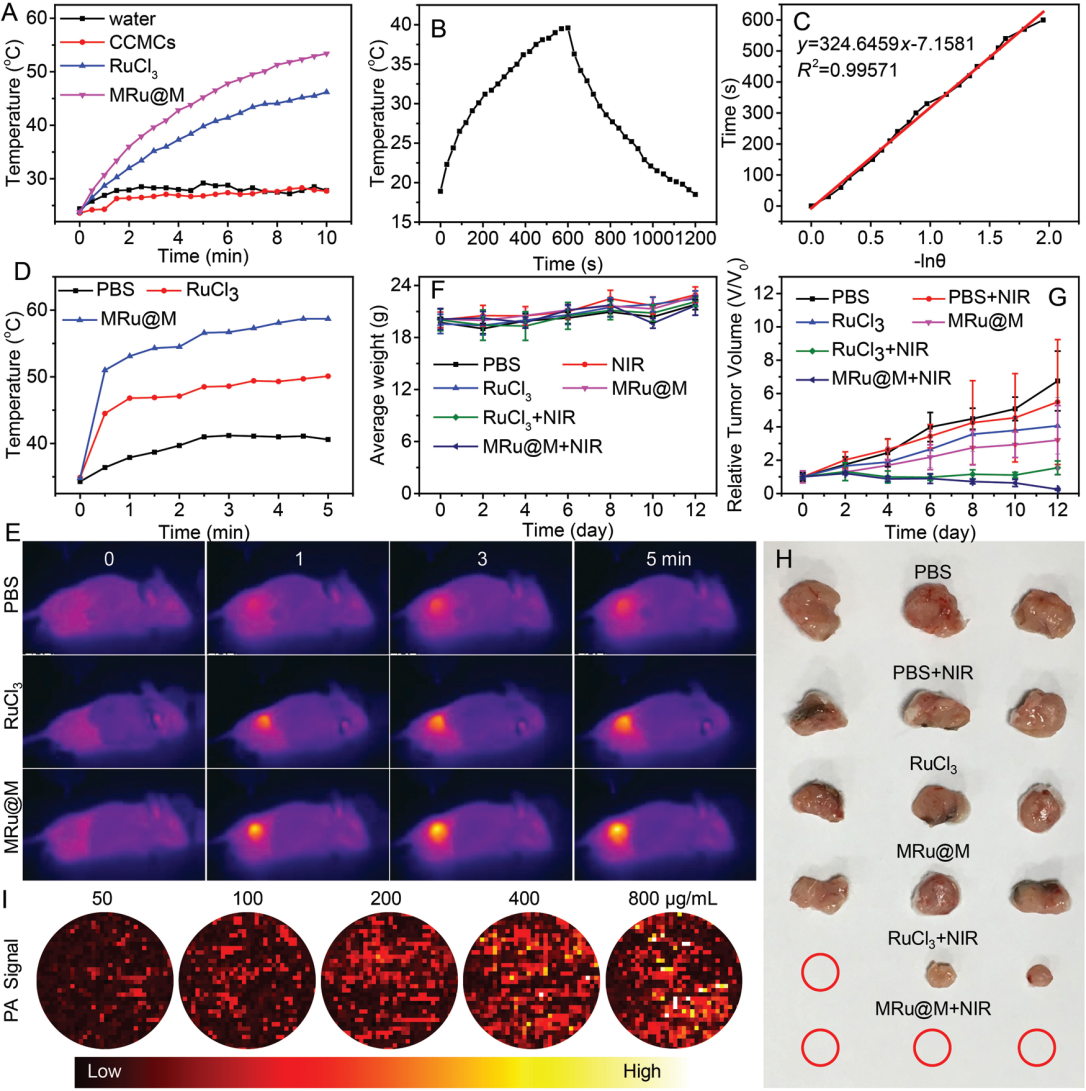
A) Temperature curves of water, CCMCs, RuCl_3_, and MRu@M solutions upon the NIR irradiation at power density of (0.8 W cm^−2^). B) The temperature profile of MRu@M solution irradiated with 808‐nm laser, followed by natural cooling after the laser was turned off, C) determination of the system time constant using linear regression of the cooling profile shown in (B). D) Temperature curves and E) thermal photo images of the mice in groups of PBS, RuCl_3_, and MRu@M upon the NIR irradiation at power density of (0.8 W cm^−2^). F) Variation of average weight of the mice after photothermal therapy mediated with different samples. G) Variation of relative tumor volume after different treatments. H) Photographs of tumor in different groups after treatment. I) Images of PA signal of MRu@M nanoparticles at different concentration.

Fluorescent materials have attracted great interests in recent years due to their important applications, such as fluorescent dyes,^40^ luminescence probes,^41^ optical bioimaging,^42^ chemical sensing,^43^ and electrochromic display.^44^ Organic fluorescent materials featured with low molecular weight and good solubility have been extensively explored during last decades.^45^ However, their applications are limited by the poor light stability and the broad emission bandwidth.^46^ In comparison, many metal complexes exhibit fascinating fluorescence properties, for example, sharp emission bands and extremely long luminescence lifetimes.^47^ Lanthanide ions are among the privileged emissive species, which are particularly attractive for visualization over the molecular events of biological systems.^48^ However, their fluorescence stability is hampered by the interference of water molecules as a competitive coordination ligand for lanthanide ions.^49^ The poor selectivity to target site of metal ion‐based fluorescent probe is another important challenge against their in vivo application.^2^ Here, MEu‐TTA@M nanoparticles were prepared from Eu‐TTA (complex of Eu^3+^ and 2‐thenoyltrifluoroacetone; Figure S21, Supporting Information). As shown in **Figure** 4A, the fluorescent property of Eu‐TTA presented poor tolerance to aqueous condition, as evidenced by the marked decline of fluorescence intensity in water compared with that detected in the good solvent of ethanol. Compared to free Eu‐TTA with identical concentration, MEu‐TTA@M displayed enhanced fluorescence by 3.2‐folds and the decay time extended 2.4‐folds in aqueous solution (Figure 4B). MEu‐TTA@M in aqueous medium gave even better fluorescence performance than Eu‐TTA in ethanol. It was evident that the fluorescence property of Eu‐TTA can be largely enhanced and stabilized under the protection of cytomembrane. In addition to this improvement, the cellular uptake efficiency can be concurrently enhanced possibly owing to the endocytosis pathway of MEu‐TTA@M nanoparticles. As shown in Figure 4C,D, red fluorescence was clearly detected in the MEu‐TTA@M treated cells, while it was barely detectable in the Eu‐TTA‐treated cells. These results demonstrated that the complexation with CCMCs can effectively improve the stability of metal conjugates in aqueous solution and facilitate their intracellular delivery. This advantage ought to be amplified in vivo when the specific targeting ability of CCMCs is taken into consideration.

**Figure 4:**
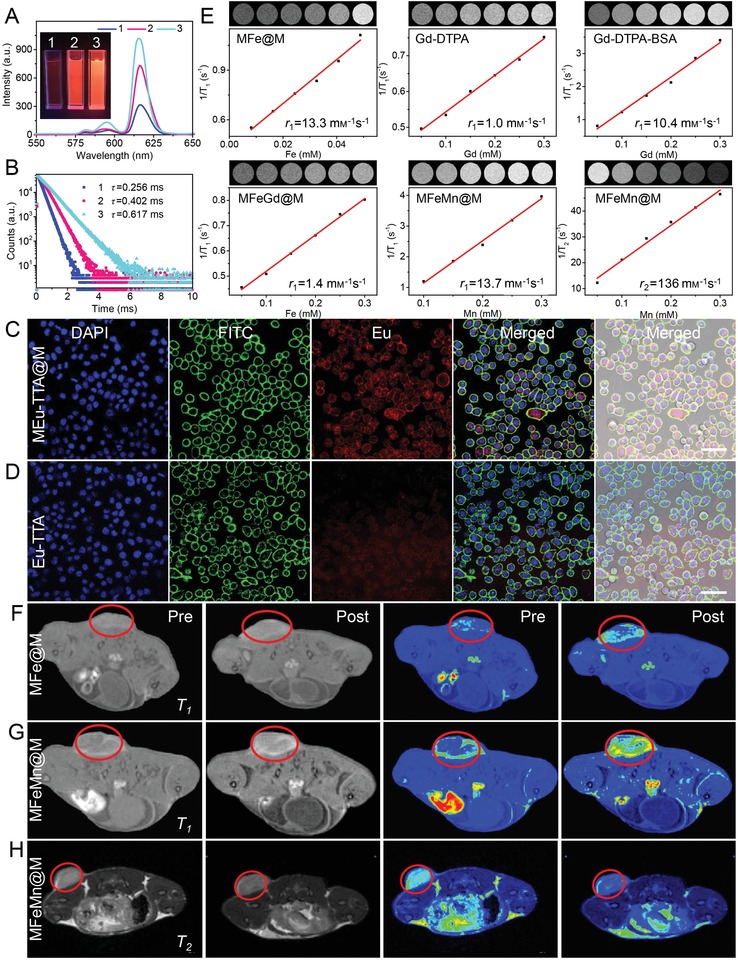
A) Photographs (insert) and fluorescence intensity of Eu‐TTA and MEu‐TTA@M. B) The decay curves of Eu‐TTA and MEu‐TTA@M. (1, Eu‐TTA in aqueous solution; 2, Eu‐TTA in ethanol; 3, MEu‐TTA@M in aqueous solution). C,D) Cellular internalization of MEu‐TTA@M (C) and Eu‐TTA (D) in 4T1 cells. Scale bar: 50 µm. E) *T_1_*‐MR images (insert) and plots of *T_1_* relaxation rate (1/*T_1_*) in the solutions of MFe@M, Gd‐DTPA, Gd‐DTPA‐BSA, MFeGd@M, and MFeMn@M as well as *T_2_*‐MR images (insert) and plots of *T_2_* relaxation rate (1/*T_2_*) in MFeMn@M solution. F) 2D axial *T_1_*‐weighted spin‐echo MR images and the corresponding color coding of MR images before (pre) and after (post) intravenous injection of MFe@M and G) MFeMn@M. H) 2D axial *T_2_*‐weighted spin‐echo MR images and corresponding color coding of MR images before (pre) and after (post) intravenous injection of MFeMn@M.

MRI has been widely used in disease diagnosis, which can be attributed to its high resolution and noninvasive characteristics.^50^ Metal ions play a vital role in MRI, such as iron (Fe),^51^ manganese (Mn),^52^ and gadolinium (Gd).^53^, ^54^ However, small‐molecule imaging reagents face some major challenges, such as weak in vivo signals, short half‐lives, and susceptibility of clearance from body.^54^, ^55^ Macromolecular contrast agents prepared from the conjugation with polymeric backbones lack targeting function, leading to poor imaging performance accompanied with side effects. Here, we used cytomembranes to deliver these magnetic metal ions in attempt to offer improved tumor imaging. At the magnetic field strength of 4.7 T, the *r*_1_ relaxivity of MFe@M nanoparticles was 13.3 mm ^−1^ s^−1^, approximately 13‐fold higher than the commercial agent of Gd‐DTPA (1.0 mm ^−1^ s^−1^) and 1.3‐fold (10.4 mm ^−1^ s^−1^) than macromolecular agent of Gd‐DTPA‐BSA (Figure 4E). We also prepared multi‐metal MFeGd@M and MFeMn@M nanoparticles (Figure S22, Supporting Information). As shown in Figure 4E, the *r*_1_ relaxivity of MFeMn@M was 13.7 mm ^−1^ s^−1^, slightly higher than MFe@M. Interestingly, the *r*_2_ relaxivity of MFeMn@M nanoparticles reached 136 mm ^−1^ s^−1^, which was higher than most of commercial contrast agents.^56^ Next, these complexes were investigated for in vivo tumor imaging. 4T1‐tumor‐bearing mice were intravenously injected with Gd‐DTPA, Gd‐DTPA‐BSA, MFe@M, and MFeMn@M, respectively. The mice were subjected to MRI imaging at the predetermined interval. MFe@M and MFeMn@M nanoparticles accumulated in tumor site and offered a clear *T_1_*‐weighted imaging of tumors (Figure 4F,G), which was better than Gd‐DTPA (Figure S23, Supporting Information) and Gd‐DTPA‐BSA (Figure S24, Supporting Information). Moreover, MFeMn@M nanoparticles provided favorable in vivo *T_2_*‐weighted imaging, which adumbrated an appealing methodology to develop multimodal MRI agents (Figure 4H). These results demonstrated that our strategy can readily deliver single and more metal ions, thus offering a versatile platform with adjustable compositions for complicated applications.

In summary, we developed a new concept of cytomembrane‐mediated biospecific transport of metal ions without using any other materials. CCMCs can readily complex with metal ions via two‐step preparation to provide hybrid nanomaterials. Relying on innate biofunctions of CCMCs, the transport of metal ions was featured with excellent biocompatibility, low immunogenic risk, and particularly specific tumor‐targeting ability. As a proof‐of‐concept demonstration, metal ions, such as ruthenium, europium, iron and manganese, were complexation with CCMCs, offering a series of tumor‐targeted nanomaterials capable of PTT, MRI, PAI, PTI, or fluorescent imaging with improved performances. Interestingly, this methodology provides opportunities to accommodate multiple metal ions for concurrent delivery, favoring complicated applications. The special structure of cell membrane also allows the effective incorporation of small‐molecular drugs to provide a novel chemotherapy nanoplatform. This new concept exhibited superior advantages over the common approaches used for the transport of metal ions and showed promising potentials in biomedical applications.

## Conflict of Interest

The authors declare no conflict of interest.

## Supporting information

Supplementary
