# Supplementary material for: Cytomembrane‐Mediated Transport of Metal Ions with Biological Specificity
Source: Adv Sci (Weinh). 2019 Jul 1;6(17):1900835. doi: 10.1002/advs.201900835 (PMC6724363; doi:10.1002/advs.201900835)
Supplement: Supplementary file 1 — Supplementary [file ADVS-6-1900835-s001.pdf]

Copyright WILEY-VCH Verlag GmbH & Co. KGaA, 69469 Weinheim, Germany, 2019.

Supporting Information

for *Adv. Sci.,* DOI: 10.1002/advs.201900835

Cytomembrane-Mediated Transport of Metal Ions with Biological Specificity

Ming-Kang Zhang, Jing-Jie Ye, Chu-Xin Li, Yu Xia, Zi-Yang Wang, Jun Feng, and Xian-Zheng Zhang*

WILEY-VCH

Copyright WILEY-VCH Verlag GmbH & Co. KGaA, 69469 Weinheim, Germany, 2019.

Supporting Information

Cytomembrane Mediated Transport of Metal Ions with Biological Specificity

*Ming-Kang Zhang, Jing-Jie Ye, Chu-Xin Li, Yu Xia, Zi-Yang Wang, Jun Feng** *and Xian-Zheng Zhang*

M.-K. Zhang, *J.-J. Ye, C.-X. Li, Y. Xia, Z.-Y. Wang*, Prof. Dr. J. Feng, Prof. Dr. X.-Z. Zhang

Key Laboratory of Biomedical Polymers of Ministry of Education & Department of Chemistry

Wuhan University

Wuhan 430072, P.R. China

E-mail: fengjun@whu.edu.cn

Table of Contents for Supporting Information:

Experimental Section…………………………………………………………………...5

Figure S1. Variation of zeta potential of MFe with increasing dose of iron ion………………………………………………………………………….………..12

Figure S2. Hydrodynamic diameter (A) and zeta potential (B) of MFe and MFe@M in ultrapure water…………………...13

Figure S3. Variation of hydrodynamic diameters with time of MFe@M nanoparticles in PBS and DMEM...14

Figure S4. Hydrodynamic diameters of MFe nanoparticles in water, PBS and the cell culture medium of DMEM...15

Figure S5. UV-Vis spectra of CCMCs and the composites consisting of metal ions and CCMCs...16

Figure S6. Hemolysis test of $FeCl_3$ and MFe@M nanoparticles at different Fe concentrations (5, 10, 20, 40 μg/mL) in PBS..17

Figure S7. Cytotoxicity assay of MFe@M nanoparticles in NCTC 1649 and 4T1 cells at different concentrations...18

Figure S8. Two-weeks blood biochemistry analysis toward the MFe@M treated mice. (TP: total protein, ALB: albumin, GLO: globulin, TBIL: total bilirubin, ALT: alanine

aminotransferase, AST: aspartate aminotransferase, GGT: γ-glutamyl transpeptidase, UREA: urea, GLU: glucose)..19

Figure S9. Two-weeks blood routine analysis toward the MFe@M treated mice. (Major items: RBC: red blood cell, WBC: white blood cell, PLT: platelets, HCT: hematocrit, MCV: mean corpuscular volume, HGB: hemoglobin)..20

Figure S10. (A) Western blot analysis of CD47 protein in CCMCs and MFe@M nanopratocles. (B-D) Flow cytometry of (B) live 4T1 cells, (C) apoptotic 4T1 cells and (D) MFe@M after the treatment with PE-CD47 for staining CD47 and Annexin V-FITC for staining phosphatidylserine, respectively. The apoptotic 4T1 cells were acquired by co-incubating 4T1 cells with carbonyl cyanide 3-chlorophenylhydrazone.. 21

Figure S11. Flow cytometry of different cells incubated with MFe@M nanoparticles. The cells without MFe@M treatment served as the negative control........................22

Figure S12. SDS-PAGE protein analysis of MFe, MFe@M and CCMCs..................23

Figure S13. TEM image of MFe@M-DOX..24

Figure S14. Variation of average weight of the mice after the intravenous injection of DOX-containing samples..25

Figure S15. H&E staining images of major organ slices isolated at 14th day after the intravenous injection of DOX-containing samples..26

Figure S16. TEM image of MRu@M...27

Figure S17. (A) Photo image of $RuCl_3$, CCMCs and MRu@M.................................28

Figure S18. Thermal images of different solutions recorded with time by an IR camera during laser irradiation (808 nm laser, 0.8 W/cm^2)..29

Figure S19. H&E staining images of major organ slices after photothermal therapy..30

Figure S20. *In vitro* PA signals of MRu@M solutions with different concentrations...31

Figure S21. TEM images of MEu-TTA@M..32

Figure S22. TEM images of MFeMn@M (A) and MFeGd@M (B)...........................33

Figure S23. *In vivo* T_1-weighted MR images of 4T1-tumor-bearing mice before (left) and after (right) intravenous injection of Gd-DTPA...34

Figure S24. *In vivo* T_1-weighted MR images of 4T1-tumor-bearing mice before and after intravenous injection of Gd-DTPA-BSA..35

Experimental Section

Materials. Ferric chloride ($FeCl_3$), manganese chloride ($MnCl_2$), gadolinium chloride ($GdCl_3$), ruthenium chloride ($RuCl_3$), copper chloride ($CuCl_2$), and chromic chloride ($CrCl_3$) were purchased from Sinopharm Chemical Reagent Co. (China). Doxorubicin (DOX) was provided by Dalian Meilun Biotech Co., Ltd. Dulbecco's modified eagle medium (DMEM), roswell park memorial institute (RMPI) 1640 medium, trypsin, penicillin streptomycin, fetal bovine serum (FBS), 3-(4,5-dimethyl-2-thiazolyl)-2,5-diphenyl-2-H-tetrazolium bromide (MTT), CellMask Green Plasma Membrane Stain and phosphate-buffered saline (PBS) were purchased from Invitrogen. Molecular probe 2-(4-Amidinophenyl)-6-indolecarbamidine dihydrochloride (DAPI), Hoechst33342, membrane protein extraction kit, phenylmethanesulfonyl fluoride (PMSF) and 1,1-dioctadecyl-3,3,3,3-tetramethylindotricarbocyaine (DIR) were obtained from Beyotime Biotechnology (China). Annexin V-FITC/PI apoptosis and necrosis detection kit (Cat No.40302ES60) was purchased from Yeasen Co., Ltd (Shanghai,China). All other reagents and solvents were of analytical grade and used as received.

Apparatus. Transmission electronic microscopy (TEM) acquired by JEOL-2100 (JEOL Ltd., Japan). The hydrodynamic size, the ζ-potentials and the stability were measured on Nano-ZS ZEN3600 (Malvern Instruments, UK) at 25°C. The absorbance spectra were measured by UV−vis spectrophotometer (Lambda Bio40, PerkinElmer,

USA). The cytotoxicity was measured by microplate reader (Bio-Rad, model 550, USA). The fluorescence images of cells internalization were acquired by a confocal laser scanning microscope (C1−Si, Nikon, Japan and PerkinElmer Ultra VIEW VoX). Quantitative fluorescent was analyzed by Flow cytometry (BD FACS Aria TM III). The temperature variation under different condition was monitored by an FLIR A × 5 camera (FLIR Systems AB, Sweden). Tumor detection experiments (*in vivo* imaging) were observed by IVIS imaging systems (PerkinElmer). *In vitro* and *in vivo* magnetic resonance imaging assays were performed by a 4.7T small animal MRI system (Bruker Biospin, USR47/30, Billerica, MA). Blood biochemistry and blood routine were tested by Blood Biochemistry Analyzer (MNCHIP POINTCARE) and Auto Hematology Analyzer (MC-6200VET).

Preparation of cancer cell membrane cracks. The collection method of cell membrane cracks was according to previous reported. 4T1 and CT26 cells were incubated in roswell park memorial institute (RMPI) 1640 medium containing 10% FBS and 1% antibiotics (penicillin-streptomycin). To obtain membrane cracks, cells were incubated in cell culture dishes with 7.5 cm in diameter, then digested with trypsin, and isolated by centrifugation treatment at 1200 rpm for 3 min. Then the collected cells were washed by PBS buffer (pH=7.4) and centrifuged again. The obtained cell pellets were suspended in a hypotonic lysing buffer containing membrane protein extraction reagent and phenylmethanesulfonyl fluoride (PMSF), then incubated in ice bath for 15 min. After that, the collected cells in the above solution were broken repeatedly using a freeze-thaw method followed by

centrifugation at 700 g for 10 min at 4 ^{o}C. Then, the supernatant was subjected to further centrifugation at 14,000 g for 30 min to collect the cell membrane cracks. The membrane products were lyophilized overnight, weighed and stored at -80 °C.

Preparation of MFe and MFe@M. Cancer cell membrane crack was dispersed in deionized water at a concentration of 1 mg/mL by ultrasonic. 20 μL ferric chloride solution (10 mg/mL) was added dropwise to the cell membrane solution (1 mg/mL), then repeated vortexing and ultrasound. The mixture solution was performed centrifugation at a rotate speed of 12000 rpm for 30 min, the precipitate was collected and named as MFe.

MFe was dispersed in deionized water as a concentration of 2 mg/mL and added dropwise to the cytomembrane solution (2 mg/mL) with constantly vortexing and ultrasound until the mixture became clear and transparent. Then the mixture solution was physically extruded by an Avanti mini extruder. The MFe@M nanoparticles were finally obtained by centrifuging at 12000 rpm for 30 min and washing repeatedly.

Hemolysis Test: Blood cells were collected from female BALB/c mice and incubated with different concentration (5, 10, 20, 40 μg/mL) of Fe^{3+} in centrifuge tubes at 37 °C for 4 h. Then the samples were centrifuged at 1200 rpm for 3 min and the supernatant was collected to measure the hemolysis rate. Briefly, 100 μL of supernatant of different samples were added to the 96-well plate, and every group has five parallel samples, then measured the absorbance at 541 nm by a microplate reader (Bio-Rad, model 550, USA) The blood cells dispersed in distilled water and PBS

were set as the positive and negative control, respectively. The hemolysis rate was calculated as ($OD541_{sample}$-$OD541_{negative}$/$OD541_{positive}$-$OD541_{negative}$) × 100%.

Cell Culture. Mouse breast cancer cells (4T1) were incubated in RPMI 1640 medium with 10% FBS and 1% antibiotics (penicillin–streptomycin, 10000 U/mL). Mouse melanoma cells (B16), African green monkey fibroblast (COS7) cells, human hepatoma carcinoma cells (HepG2) and mouse normal hepatic cells (NCTC 1469) were incubated in DMEM with 10% FBS and 1% antibiotics (penicillin–streptomycin, 10000 U/mL). The cells were cultured in a humidified atmosphere with 5% CO_2 at 37 ^oC.

In Vitro Cytotoxicity Assay. The cytotoxicity was estimated in 4T1 and NCTC1469 cells by MTT assay. In brief, 4T1 and NCTC1469 cells were seeded in a 96-well plate at a density of $6×10^3$ cells per well and cultured in 100 μL of RMPI 1640 and DMEM medium. After 24 h incubation, the culture medium was removed and replaced with fresh RPMI 1640 and DMEM medium, and gradient concentrations of MFe@M were added into each well. After 24 h co-incubation, 20 μL of MTT (5 mg/mL) was added and incubated for another 4 h. After that, the medium was removed and 150 μL DMSO was added. The absorbance at 570 nm (OD570) was measured by a microplate reader (Model 550, Bio-Rad, USA). The relative cell viability was calculated as ($OD570_{sample}$/$OD570_{control}$) × 100%, where $OD570_{control}$ was obtained from untreated cells and $OD570_{sample}$ was obtained after different treatments.

Cell Internalization. RAW 264.7, 4T1, B16, COS7 and HepG2 cells were seeded in a glass bottom dish at a density of 1 × 10^5 cells per well, respectively. The RAW 264.7 cells were incubated with DIR labeled MFe and MFe@M, respectively. And other cells were incubated with DIR marked MFe@M (100 μg/mL) for 4 h. After that, the cultured medium was removed and the cells were washed by PBS for three times. Finally, the fluorescence images were provided by CLSM (PerkinElmer Ultra VIEW VoX).

Animals and Tumor Model. All of the animal experiments were conducted with the approval of the Institutional Animal Care and Use Committee of the Animal Experiment Center of Wuhan University (Wuhan, China) as well as the Regulations for the Administration of Affairs Concerning Experimental Animals. Female BALB/c mice (5-6 weeks) were bought from the Animal Experiment Center of Wuhan University (Wuhan, China). To obtain the tumor-bearing mice, 4T1 and CT26 cells (4 × 10^5 cells) were injected into subdermal of mice on the right back of hind leg region.

In Vivo Tumor Detection and Biodistribution. When the tumor volume reached about 100 mm^3, the mice were injected with DIR labeled MFe@M-4T1, MFe, CCMCs and MFe@M-CT26 (10 mg/kg) by caudal vein. The *in vivo* fluorescence images were observed by an IVIS imaging system (PerkinElmer) at 0, 4, 8,12, 24,48, 72, 96 h post-injection. For the tissue distribution study, the mice were euthanized after 24 h injection, tumor tissues and major organs were collected, then subjecting for *ex vivo* imaging.

In Vivo Chemotherapy Study. 4T1 tumor bearing BALB/c mice (5-6 weeks) were randomly divided into four groups (five mice in each group) and marked as: (group 1: PBS), (group 2: MFe@M), (group 3: DOX), (group 4: MFe@M-DOX). When the tumor volume reached approximately 100 mm^3, the mice were intravenously injected with PBS, MFe@M, DOX and MFe@M-DOX (10 mg/kg), respectively. The body weight and tumor volume were recorded every two days. The tumor volume was calculated as follow formula: $V = W^2 \times L/2$, where W and L represented the shortest and longest axes of tumors, respectively. Relative tumor volume was estimated as V/V_0 (V_0 represent the tumor volume before therapy).

In Vivo Photothermal Imaging. When the tumor volume reached about 100 mm^3, $RuCl_3$ and MRu@M (5 mg/kg) was intratumorally administrated into the 4T1 tumor-bearing mice. The blank control was injected with PBS buffer. After 6 h, the mice were anesthetized and the tumor areas were exposed to 808nm NIR laser at a power density of 0.8 W/cm^2 for 5 min. The infrared thermal images were provided by using a FLIR A×5 camera (FLIR Systems AB, Sweden) and the temperature of the tumor area was recorded over time.

In Vivo Photothermal Therapy Study. 4T1 tumor bearing BALB/c mice (5-6 weeks) were randomly divided into six groups (five mice in each group) and marked as: (group 1: PBS), (group 2: PBS+NIR), (group 3: $RuCl_3$), (group 4: MRu@M), (group 5: $RuCl_3$+NIR), (group 6: MRu@M+NIR). When the tumor volume reached approximately 100 mm^3, the mice of group 1 and 2 were intratumorally injected with

PBS, the mice of group 3, 5 were administrated with $RuCl_3$ and the mice of group 4, 6 were administrated with MRu@M (10 mg/kg), respectively. After 6 h, the mice of group 2, 5, 6 were subjected to the irradiation under 808nm laser at a power density of 0.8 W/cm^2 for 5 min. The body weight and tumor volume were recorded every two days. The tumor volume was calculated as follow formula: $V = W^2 \times L/2$, where W and L represented the shortest and longest axes of tumors, respectively. Relative tumor volume was determined as V/V_0 (V_0 represent the tumor volume before therapy).

In Vivo Biosafety. Healthy BALB/c mice (5-6 weeks) were randomly divided into two groups (five mice in each group) and marked as: (group 1: PBS), (group 2: MFe@M), then intravenously injected with PBS and MFe@M (10 mg/kg), respectively. 200 μL of blood was collected from heart at the predetermined intervals. The bloods were performed blood biochemistry and blood routine analysis. Liver and kidney function biochemical 9 items evaluated by a blood biochemistry analyzer (MNCHIP POINTCARE). (TP: total protein, ALB: albumin, GLO: globulin, TBIL: total bilirubin, ALT: alanine aminotransferase, AST: aspartate aminotransferase, GGT: γ-glutamyl transpeptidase, Urea, GLU: glucose). Blood routine analysis *via* an auto hematology analyzer (MC-6200VET). (Major items: RBC: red blood cell, WBC: white blood cell, PLT: platelets, HCT: hematocrit, MCV: mean corpuscular volume, HGB: hemoglobin, MCH: mean corpuscular hemoglobin, MCHC: mean corpuscular hemoglobin concentration, RDW: red blood cell distribution width).

For HE staining, the mice were euthanized with isoflurane after the treatments were finished (14 days later),then major organs (heart, liver, spleen, lung and kidney) and tumor tissues were collected for hematological and histological analysis. Major organs and tumor tissues were fixed in 4% paraformaldehyde, embedded routinely into paraffin and sectioned. Then the sections were stained by hematoxylin and eosin (H&E).

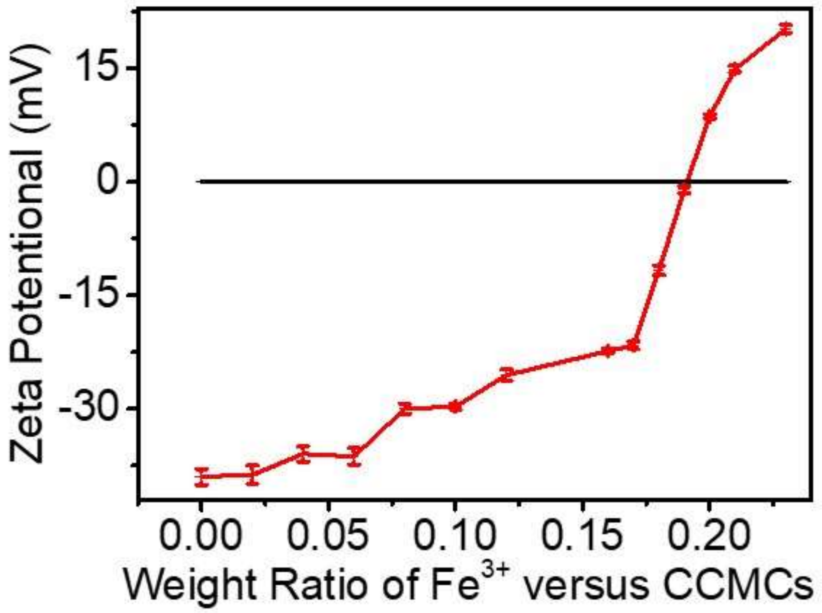

Figure S1. Variation of zeta potential of MFe with increasing dose of iron ion.

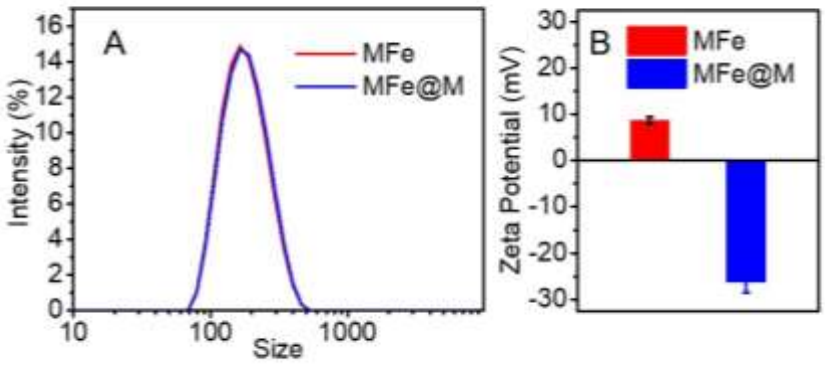

Figure S2. Hydrodynamic diameter (A) and zeta potential (B) of MFe and MFe@M in ultrapure water.

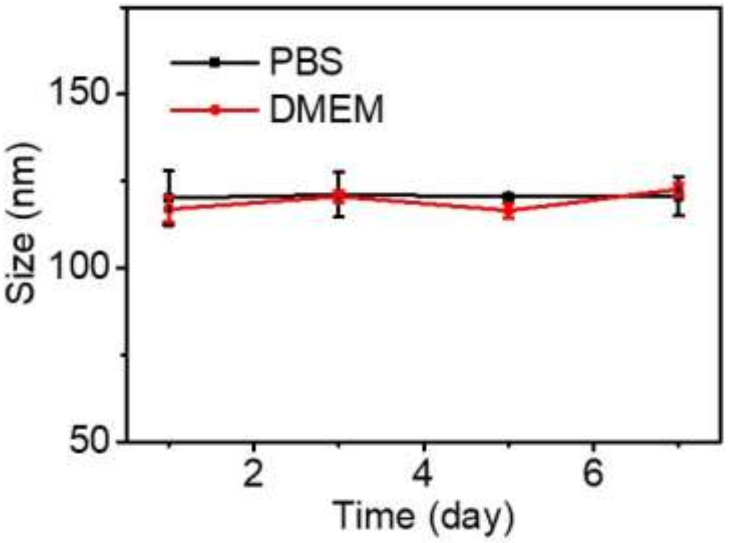

Figure S3. Variation of hydrodynamic diameters with time of MFe@M nanoparticles in PBS and DMEM.

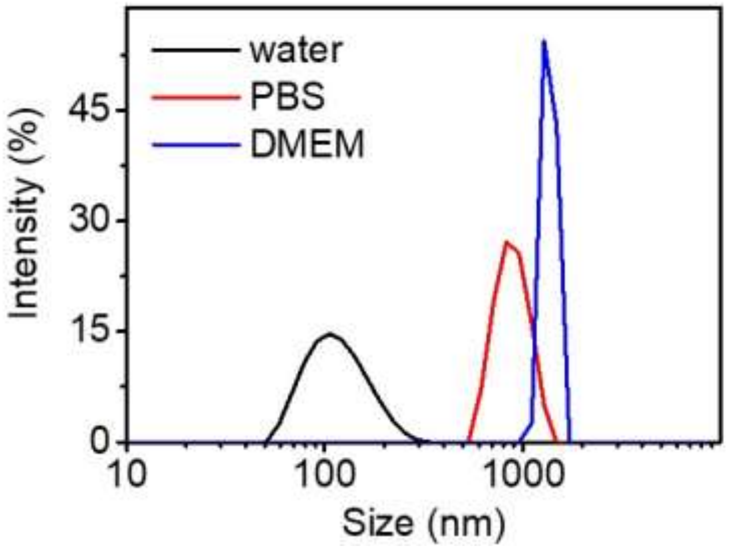

Figure S4. Hydrodynamic diameters of MFe nanoparticles in water, PBS and the cell culture medium of DMEM.

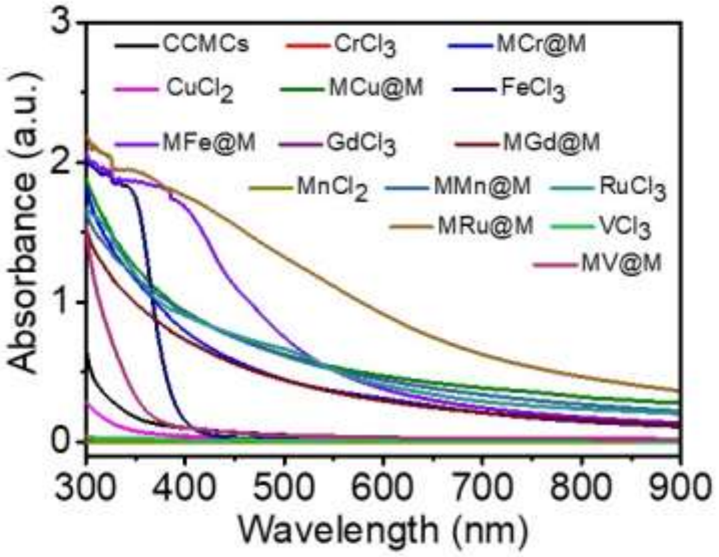

Figure S5. UV-Vis spectra of CCMCs and the composites consisting of metal ions and CCMCs.

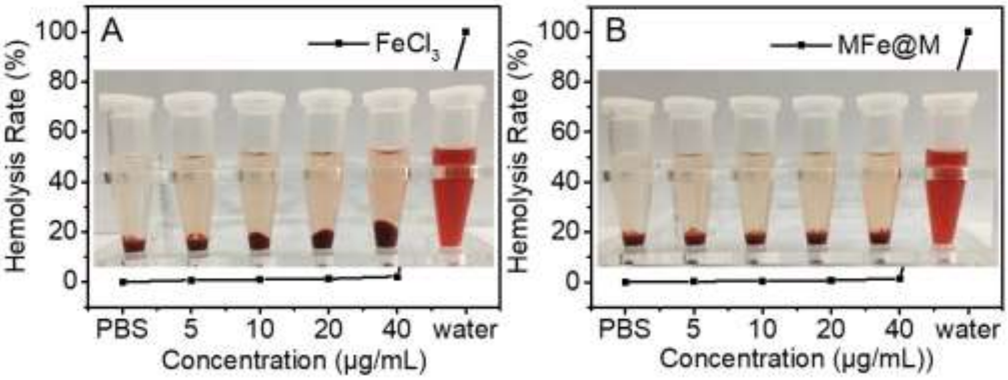

Figure S6. Hemolysis test of $FeCl_3$ (A) and MFe@M nanoparticles (B) at different Fe concentrations (5, 10, 20, 40 μg/mL) in PBS.

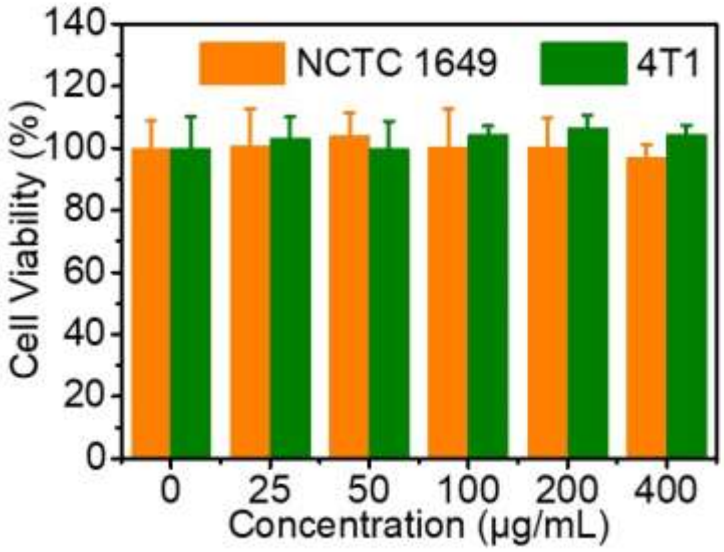

Figure S7. Cytotoxicity assay of MFe@M nanoparticles in NCTC 1649 and 4T1 cells at different concentrations.

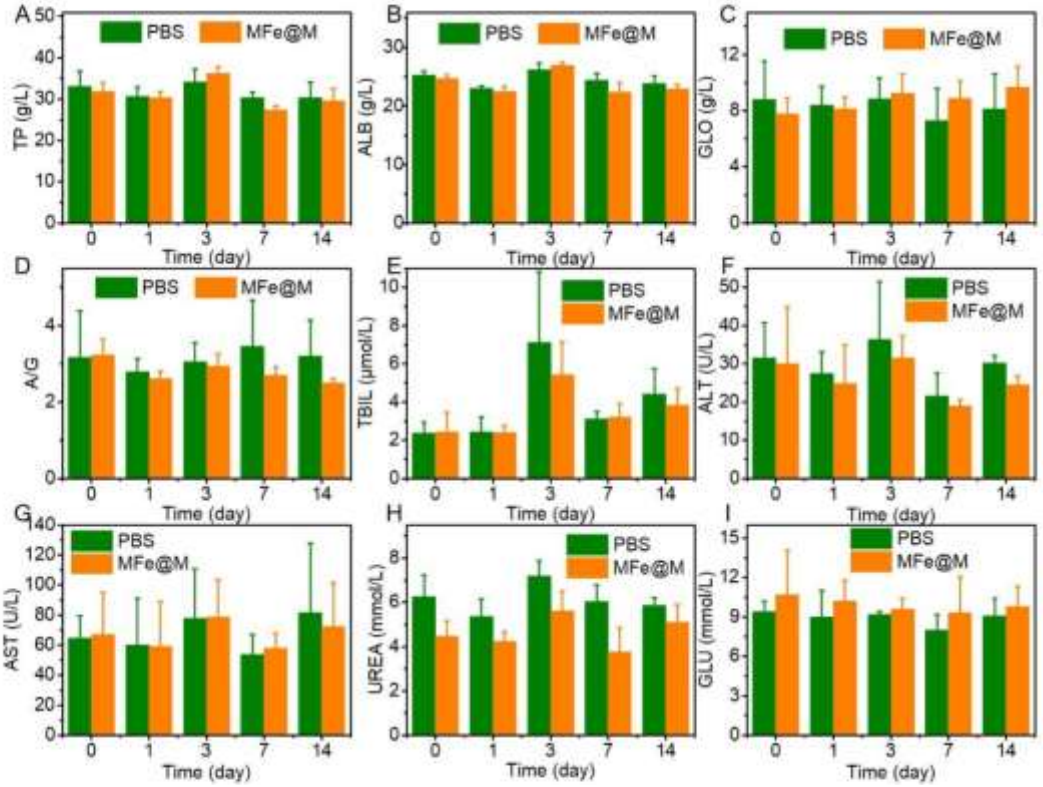

Figure S8. Two-weeks blood biochemistry analysis toward the MFe@M treated mice. (TP: total protein, ALB: albumin, GLO: globulin, TBIL: total bilirubin, ALT: alanine aminotransferase, AST: aspartate aminotransferase, GGT: γ-glutamyl transpeptidase, UREA: urea, GLU: glucose).

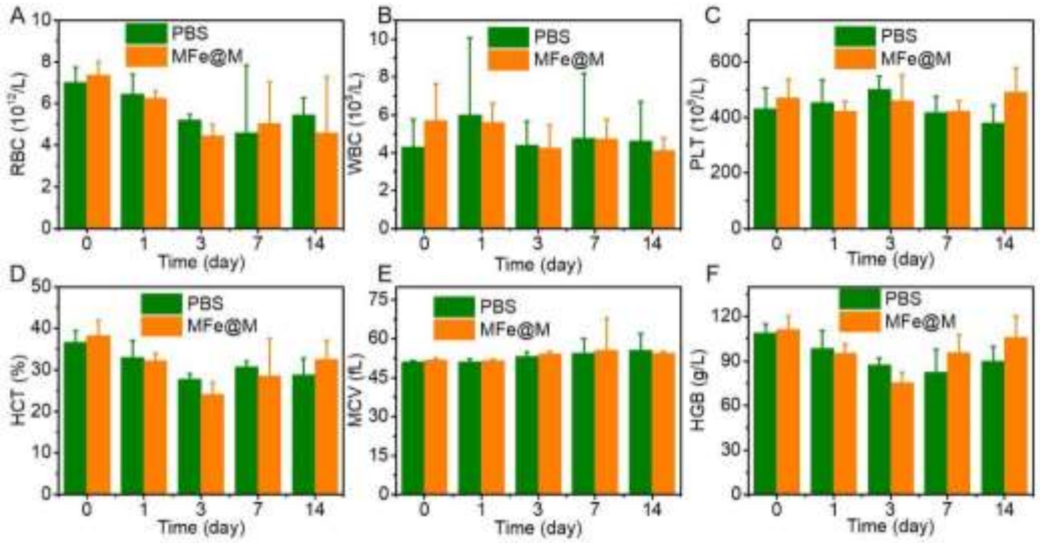

Figure S9. Two-weeks blood routine analysis toward the MFe@M treated mice. (Major items: RBC: red blood cell, WBC: white blood cell, PLT: platelets, HCT: hematocrit, MCV: mean corpuscular volume, HGB: hemoglobin).

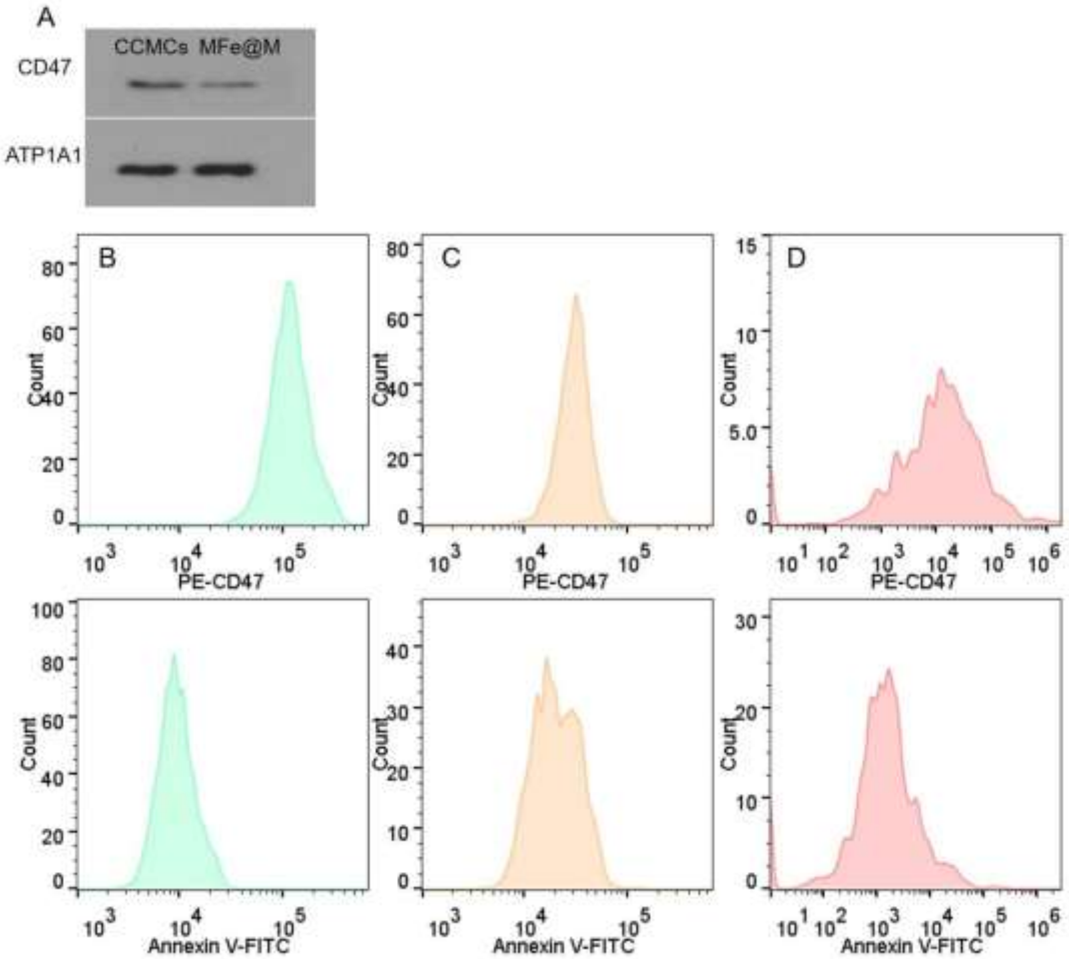

Figure S10. (A) Western blot analysis of CD47 protein in CCMCs and MFe@M nanopratocles. (B-D) Flow cytometry of (B) live 4T1 cells, (C) apoptotic 4T1 cells and (D) MFe@M after the treatment with PE-CD47 for staining CD47 and Annexin V-FITC for staining phosphatidylserine, respectively. The apoptotic 4T1 cells were acquired by co-incubating 4T1 cells with carbonyl cyanide 3-chlorophenylhydrazone.

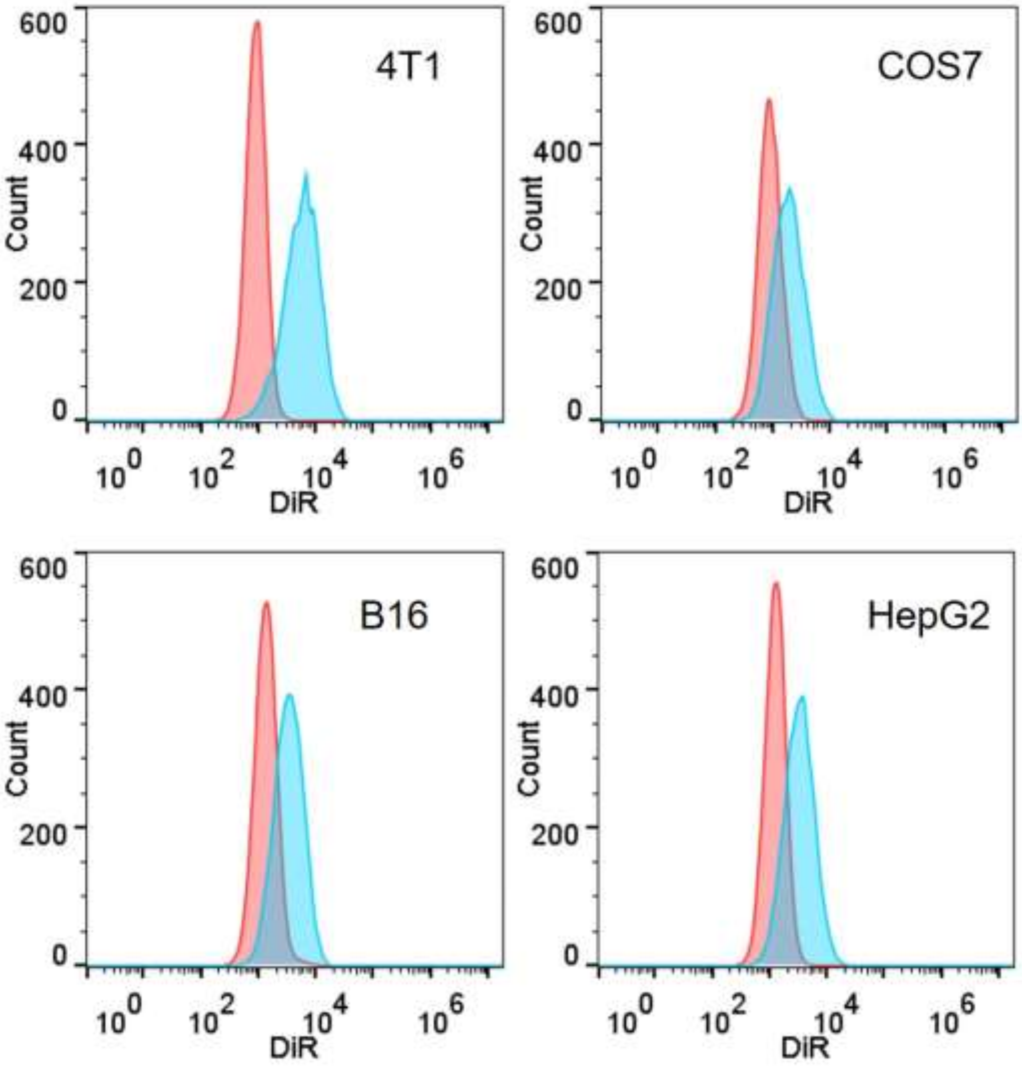

Figure S11. Flow cytometry of different cells incubated with MFe@M nanoparticles. The cells without MFe@M treatment served as the negative control.

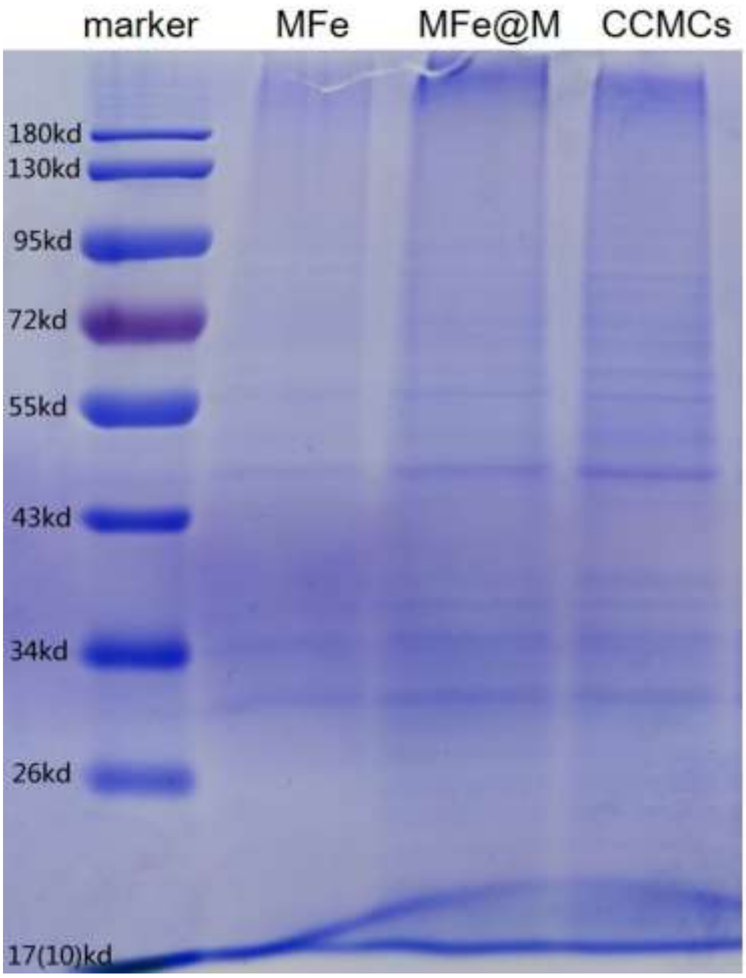

Figure S12. SDS-PAGE protein analysis of MFe, MFe@M and CCMCs.

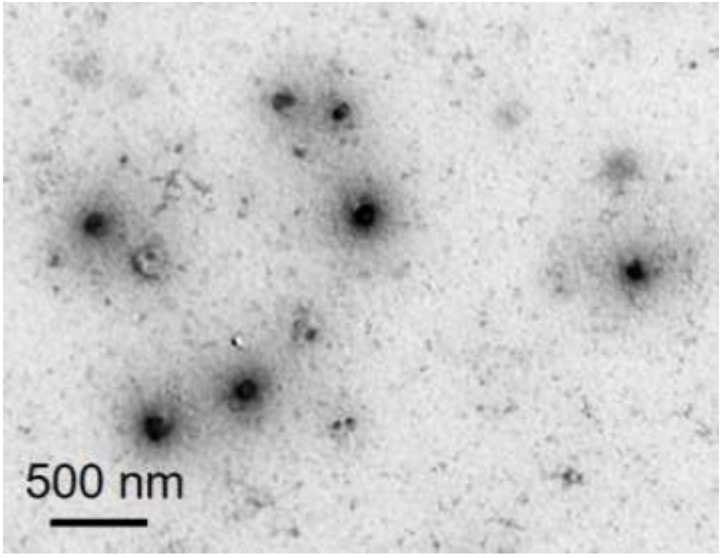

Figure S13. TEM image of MFe@M-DOX.

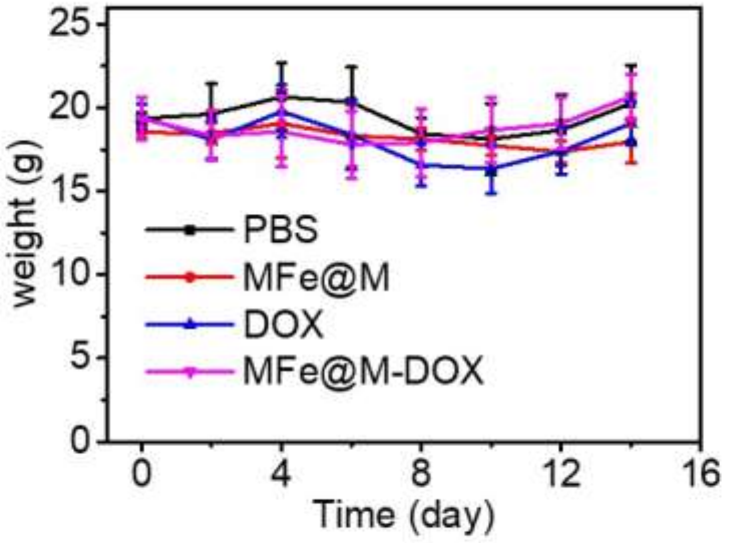

Figure S14. Variation of average weight of the mice after the intravenous injection of DOX-containing samples.

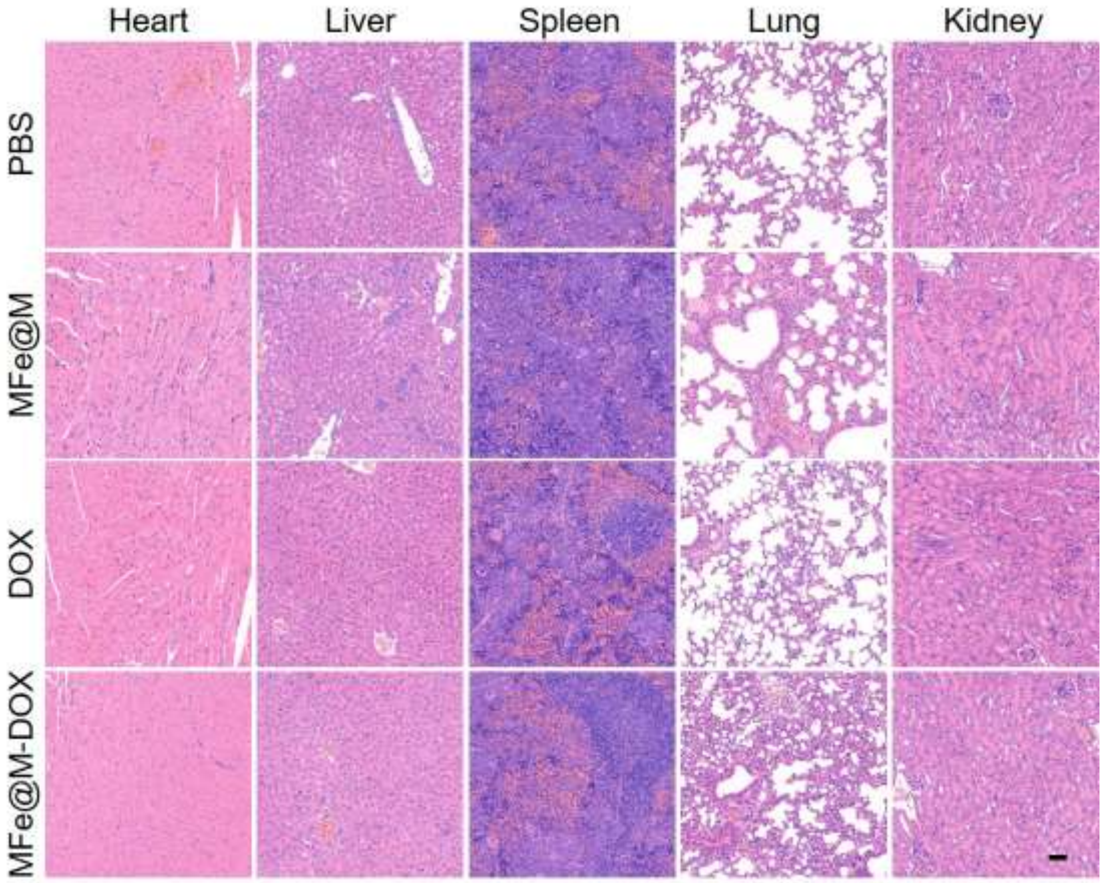

Figure S15. H&E staining images of major organ slices isolated at 14th day after the intravenous injection of DOX-containing samples. Scale bar: 50 μm.

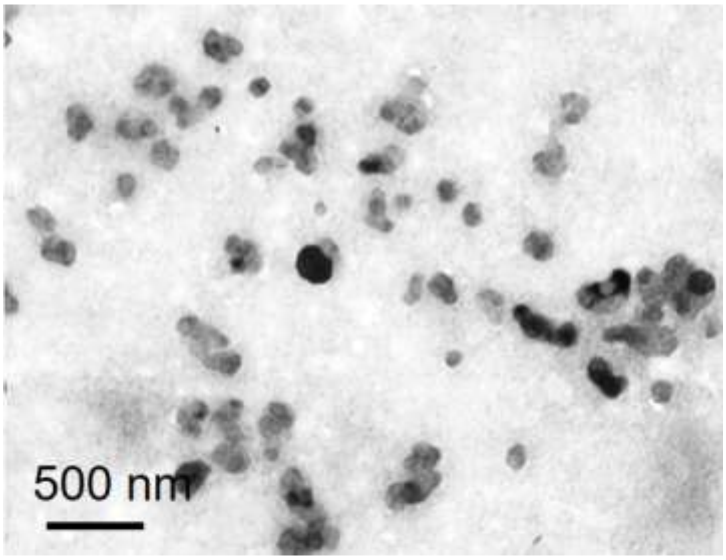

Figure S16. TEM image of MRu@M.

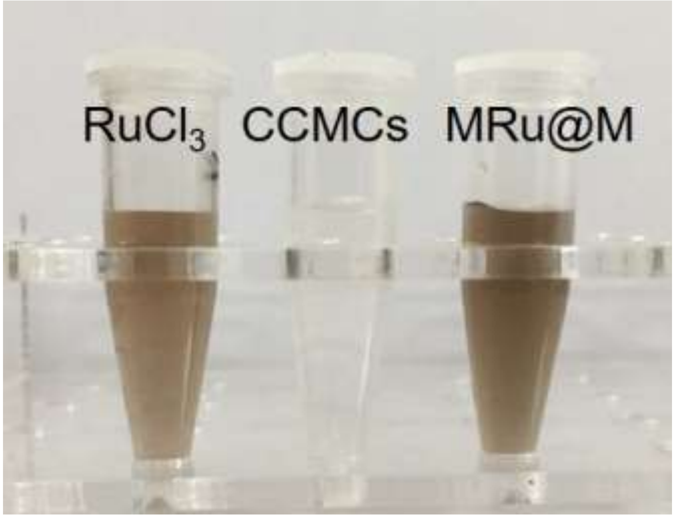

Figure S17. (A) Photo image of $RuCl_3$, CCMCs and MRu@M.

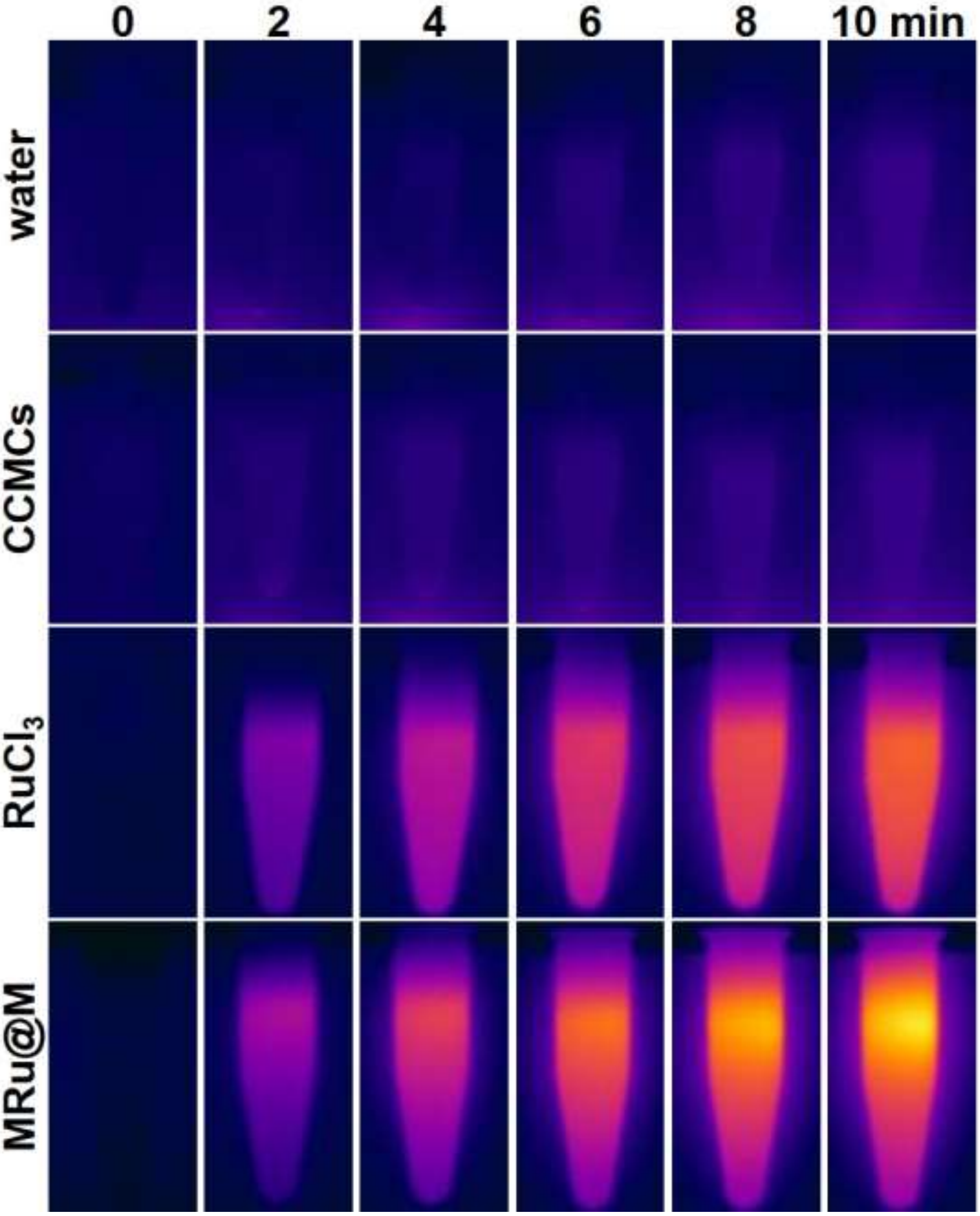

Figure S18. Thermal images of different solutions recorded with time by an IR camera during laser irradiation (808 nm laser, 0.8 W/cm^2).

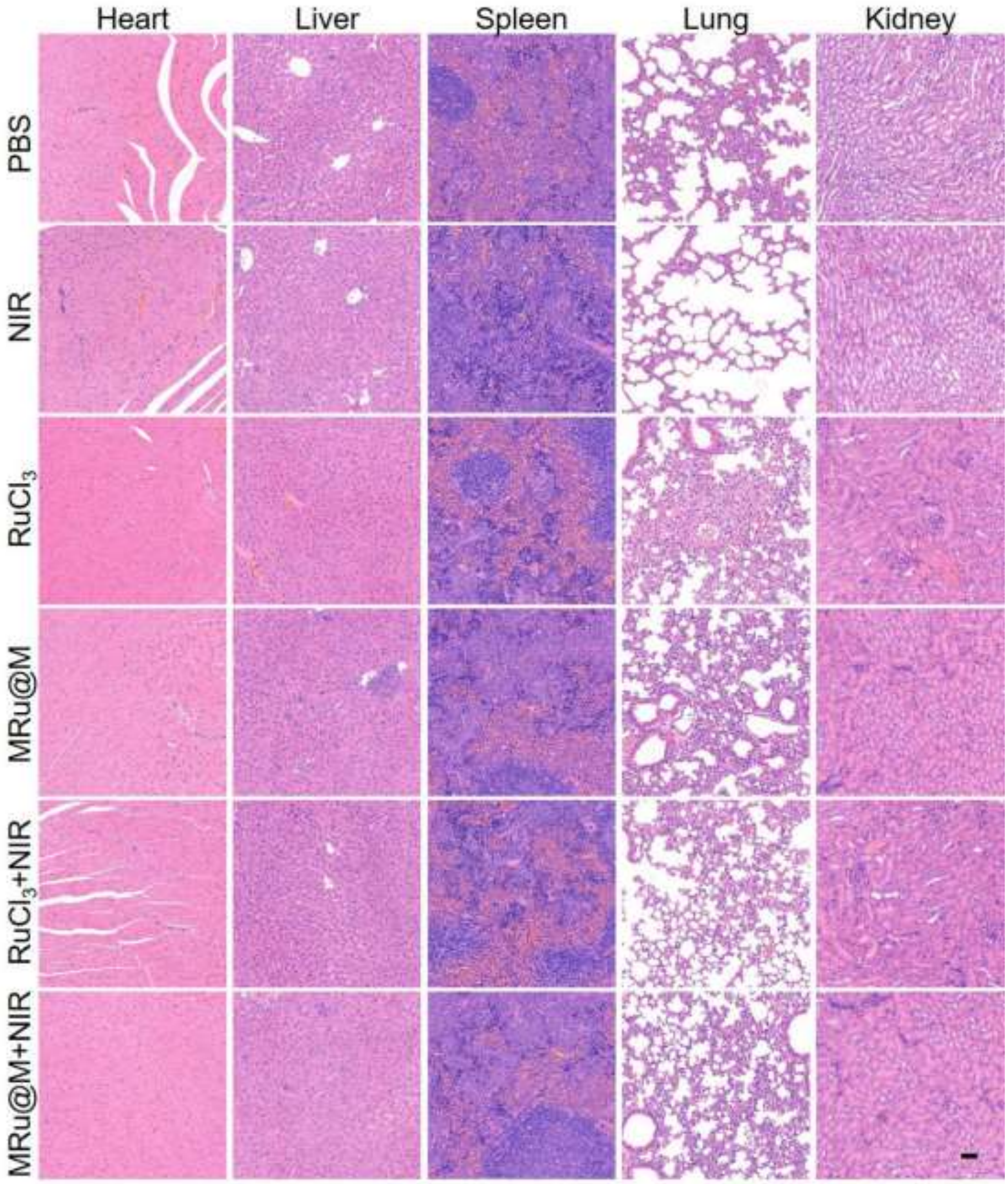

Figure S19. H&E staining images of major organ slices after photothermal therapy.

Scale bar: 50 μm.

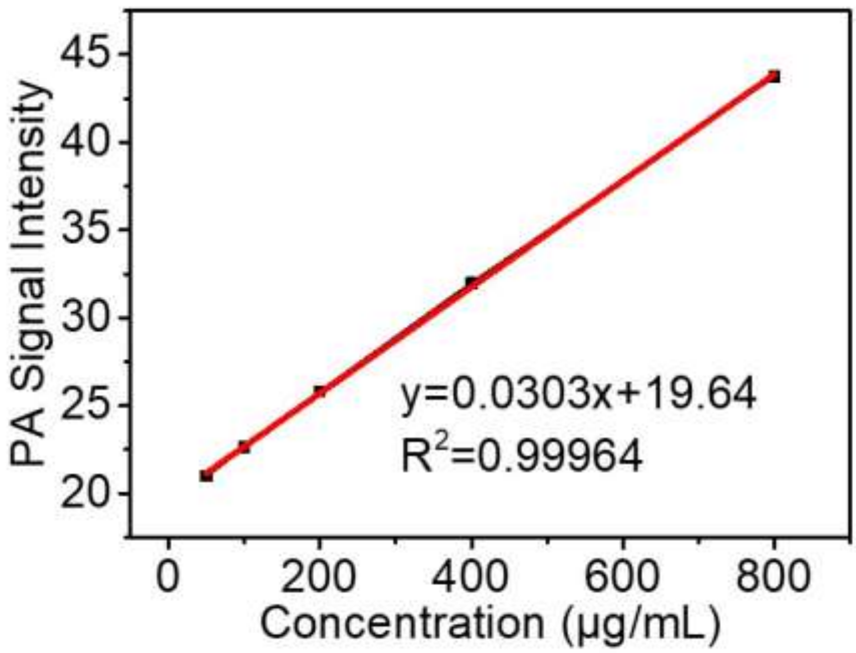

Figure S20. *In vitro* PA signals of MRu@M solutions with different concentrations.

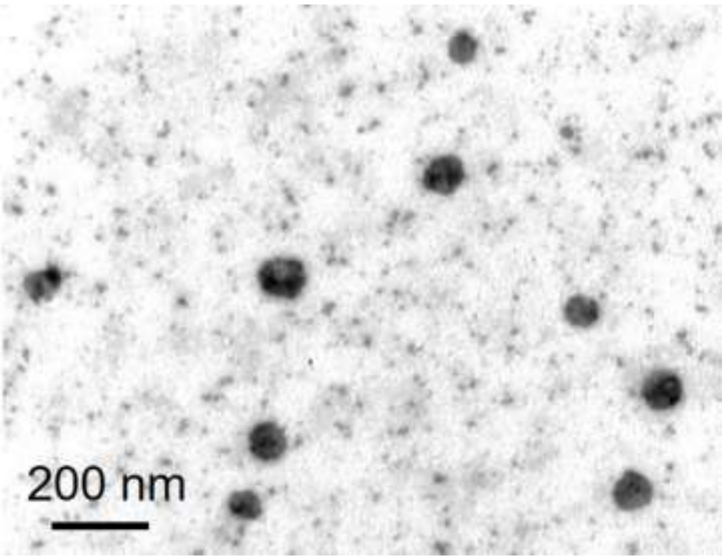

Figure S21. TEM images of MEu-TTA@M.

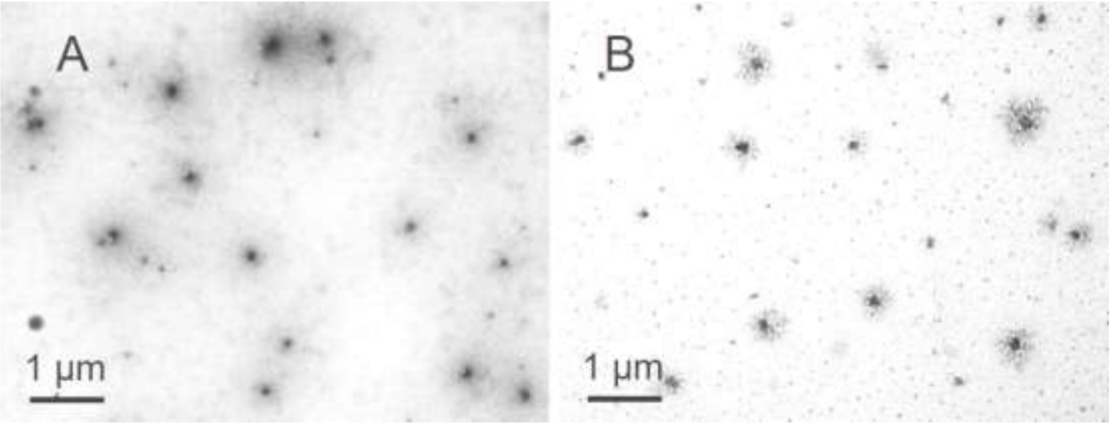

Figure S22. TEM images of MFeMn@M (A) and MFeGd@M (B).

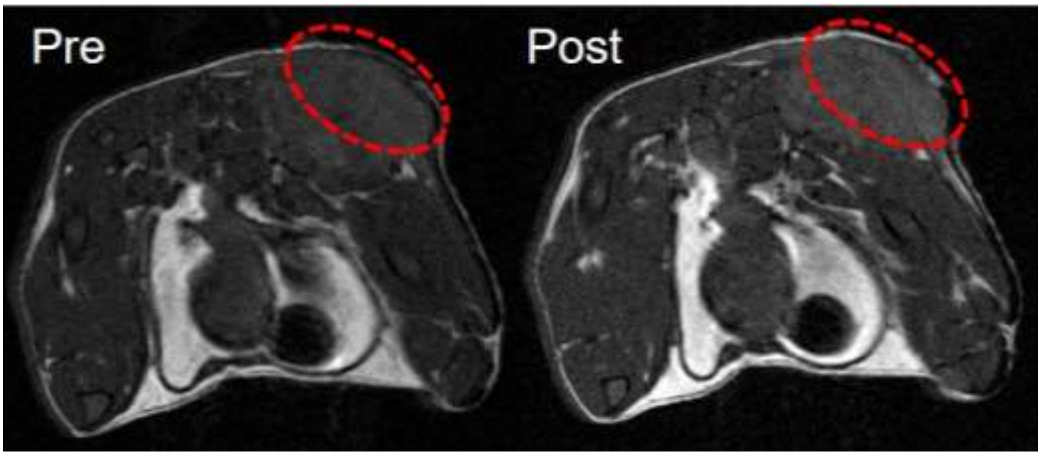

Figure S23. *In vivo* T_1-weighted MR images of 4T1-tumor-bearing mice before (left) and after (right) intravenous injection of Gd-DTPA.

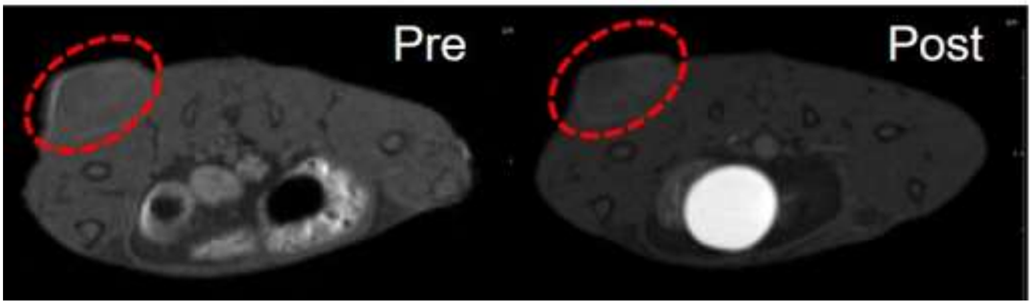

Figure S24. *In vivo* T_1-weighted MR images of 4T1-tumor-bearing mice before and after intravenous injection of Gd-DTPA-BSA.
